# Food Additive Zinc Oxide Nanoparticles: Dissolution, Interaction, Fate, Cytotoxicity, and Oral Toxicity

**DOI:** 10.3390/ijms23116074

**Published:** 2022-05-28

**Authors:** Su-Min Youn, Soo-Jin Choi

**Affiliations:** Division of Applied Food System, Major of Food Science & Technology, Seoul Women’s University, Seoul 01797, Korea; smyoun@swu.ac.kr

**Keywords:** zinc oxide (ZnO) nanoparticles (NPs), food additive, solubility, interaction, fate, oral toxicity

## Abstract

Food additive zinc oxide (ZnO) nanoparticles (NPs) are widely used as a Zn supplement in the food and agriculture industries. However, ZnO NPs are directly added to complex food-matrices and orally taken through the gastrointestinal (GI) tract where diverse matrices are present. Hence, the dissolution properties, interactions with bio- or food-matrices, and the ionic/particle fates of ZnO NPs in foods and under physiological conditions can be critical factors to understand and predict the biological responses and oral toxicity of ZnO NPs. In this review, the solubility of ZnO NPs associated with their fate in foods and the GI fluids, the qualitative and quantitative determination on the interactions between ZnO NPs and bio- or food-matrices, the approaches for the fate determination of ZnO NPs, and the interaction effects on the cytotoxicity and oral toxicity of ZnO NPs are discussed. This information will be useful for a wide range of ZnO applications in the food industry at safe levels.

## 1. Introduction

Zinc oxide (ZnO) is widely applied to the food industry as a Zn supplement, nutrient fortifier, and agricultural fertilizer due to the role of Zn in various biological functions including cell division, cell growth, immune function, enzyme activity, DNA synthesis, and protein production [1]. Zn is an essential mineral for the body and its required daily intakes for men and women are 11 mg/day and 8 mg/day, respectively [2]. The average daily Zn intake via the diet generally ranges from 8.1 to 13.5 mg/day in adults, which is sufficient for nutritional functions [3]. However, Zn deficiency frequently occurs in children and humans with dietary problem and diseases such as alcoholism, renal disease, gastrointestinal (GI) tract disorders, inflammation, and cancers [2], requiring Zn supplements or Zn-fortified foods. Zinc gluconate, sulfate, acetate, citrate, and oxide are commonly used as food additive Zn supplements [4,5]. Among them, ZnO is insoluble in water, but has a high Zn content compared with other supplements. It is registered as a generally recognized as safe (GRAS) material in the United States, suggesting its safety at actual usage levels [6].

Nanotechnology development has led to the production of nano-sized ZnO particles possessing different physicochemical properties such as small constituent particle size (1–100 nm), large surface area to volume ratio, and high reactivity compared with bulk-sized ZnO [7]. A higher efficacy of ZnO nanoparticles (NPs) as dietary supplements on the growth performance than that of conventional ZnO was demonstrated in fish [8], broilers, weaned pigs, other livestock, and Zn-deficient animal models [9,10,11]. However, high Zn intakes cause acute toxicity such as flu-like symptoms, nausea, vomiting, diarrhea, headaches, and stomachache [12,13]. Long-term exposure to high Zn levels may lead to weakened immune systems and copper deficiency [14,15]. Hence, concerns about the potential toxicity and unexpected biological responses of ZnO NPs have increased. However, current regulations for food additive ZnO do not include particle size, size distribution, nor percentage of NPs in ZnO [16,17].

In this review, recent studies on the dissolution properties and ionic/particle fates of ZnO NPs in simulated food-matrices and commercial processed foods as well as in vitro, ex vivo, and in vivo digestion systems are discussed. Methodological approaches for the fate determination of ZnO NPs in complex foods, cells, and biological systems, the characterization on the interactions between ZnO NPs and bio- or food-matrices, together with the interaction effects on cytotoxicity and in vivo oral toxicity, are summarized. These aspects should be essentially considered for a wide range of application of ZnO NPs in the food industry at safe levels.

## 2. Dissolution Properties of ZnO NPs

**Figure 1:**
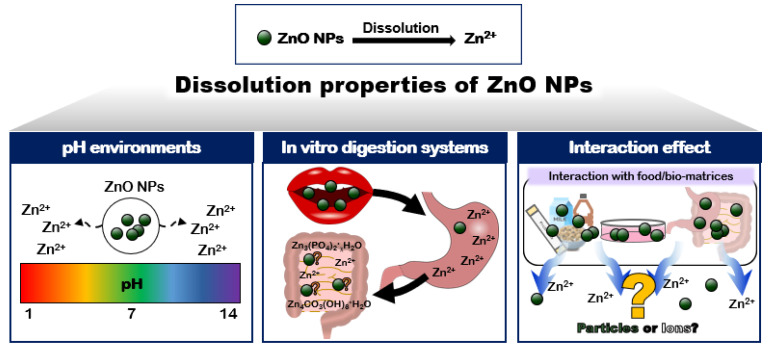
The schematic illustration of the dissolution properties of ZnO NPs effected by environmental pH, the digestion systems used, and the interactions between ZnO and the matrices.

Many reports have demonstrated that ZnO tends to dissolve under aqueous, acidic, and biological conditions [18,19,20,21]. The dissolution levels of ZnO were highly dependent on the concentrations and physicochemical properties of the materials tested. Density, agglomerate/aggregate size, and specific surface area seem to be the important physicochemical characteristics of ZnO affecting the dissolution kinetics [22]. The size-dependent dissolution of ZnO NPs has been well reported, showing that the dissolution levels increased as the particle size decreased [23,24]. These results can surely be attributed to the large specific surface area to volume ratio of the small-sized particles compared to the larger-sized ones. The effects of pH, the digestion systems used, and the interactions between ZnO and the matrices on the solubility are also crucial, which are the focus of this section (Figure 1).

### 2.1. pH Environments

ZnO is considered to be insoluble in water, but highly soluble in acidic solutions where the pH ranges from 2 to 4 [22,25]. Such low pH conditions include gastric fluid, lysosomal fluid, and some liquid beverages. Our report showed that the in vitro dissolutions of ZnO NPs (28 nm of constituent particle size, hydrodynamic diameters of ~1976 nm in distilled water (DW), 5 mg/mL) and bulk ZnO (290 nm, hydrodynamic diameters of ~3453 nm, 5 mg/mL) were 24.5%, 0.2%, and 2.8% in the simulated human gastric (pH 1.5), intestinal (pH 6.8), and plasma (5% bovine serum albumin (BSA) in phosphate buffered saline solution) fluids after 5 min to 6 h without significant differences between the particle sizes [26]. Ex vivo dissolutions of the ZnO NPs (5 mg/mL) and bulk ZnO (5 mg/mL) in the rat-extracted gastric, intestinal, and plasma fluids were ~12%, ~9%, and ~2%, respectively, after 30 min, regardless of the particle sizes [26]. In vivo dissolution of both the ZnO NPs and bulk ZnO in the gastric fluid was ~12% at 15 min post-oral administration (100 mg/kg) in rats [27]. It is interesting to note that ex vivo dissolution (~12%) of ZnO NPs in the rat gastric fluid was highly correlated with in vivo solubility (~12%) in the gastric juice after oral administration in rats. On the other hand, there was a higher in vitro solubility (24.5%) of ZnO NPs in the simulated human gastric fluid (pH 1.5) than those (~12%) in the ex vivo and in vivo rat gastric fluids, which could be related to the relatively high pH of rat gastric fluid (pH ~ 3.2) compared with that of human gastric fluid (pH ~ 1.5) [28]. In another study, a low solubility (1.9–2.1%) of two different sizes (<50 nm and <100 nm) of ZnO (0.5 mg/mL) was reported at neutral pH 7.0, whereas a high solubility (93.6–97.0%) was found at pH 1.5 after incubation for 2 h [25]. ZnO NPs (5 mg/mL) with constituent particle sizes of 78 nm and hydrodynamic diameters of 375 nm showed a solubility of ~96% in the simulated gastric fluid (pH ~1.5) [29]. Therefore, the differences in the solubility levels of the ZnO NPs among these reports are likely to be related to the concentrations and physicochemical properties of the materials tested and their aggregate/agglomerate states. Cardoso et al. reported that the dissolution kinetics of ZnO highly depend on its aggregation/agglomeration and specific surface area [22]. It is strongly likely that high agglomerate/aggregate formation and a high concentration of ZnO NPs lead to low solubility.

### 2.2. In Vitro Digestion Systems

The solubility of ZnO NPs in the GI fluids can be different from the digestion systems tested. The dissolution levels of ZnO NPs were reported as not being consistent, depending on the in vitro digestion systems applied such as a single digestive fluid (gastric, intestinal, and body fluids, separately), a three-step digestion system (gastric + intestinal + body fluids, consecutively), and a semi-closed dynamic digestion system (gastric + intestinal + body fluids, consecutively, with flow speeds using peristaltic pump) [30]. Jung et al. reported that the solubilities of ZnO NPs were ~0.1%, ~96%, and ~4% in the simulated saliva, gastric, and intestinal fluids, respectively, whereas their high solubility (~95%) in saliva, followed by gastric fluid, decreased to ~25% when a three-consecutive digestion system (saliva followed by gastric and intestinal fluids) was applied [29]. The same tendency was reported, showing a decreased dissolution level of ZnO NPs in a three-step digestion system compared with that in a single gastric fluid [31,32,33]. These results can be explained by the fact that dissolved Zn ions in the gastric fluid rapidly react with carbonate or phosphate ions massively present in the intestinal fluid, forming insoluble Zn-carbonate or Zn-phosphate [29,31]. De novo formation of particles in the intestinal fluid was demonstrated after the complete dissolution of ZnO NPs in the gastric juice, regardless of the particle type, concentration, or the presence of a food matrix by small angle X-ray scattering, [31]. Turney et al. also showed the formation of crystalline phases, Zn_4_CO_3_(OH)_6_·H_2_O or Zn_3_(PO_4_)_2_·xH_2_O (x = 2 or 4) from the Zn ions in the cell culture media containing the carbonate and phosphate anions necessary for buffer capacity [34]. The low solubility of ZnO in hard water due to the precipitation of a Zn–carbonate solid phase has been reported, suggesting the critical impact of solution chemistry on ZnO dissolution [35].

### 2.3. Interaction Effects

Another important aspect to be considered is that food additive ZnO NPs are directly added to complex foods composed of various components including proteins, carbohydrates, lipids, minerals, vitamins, and other additives. Moreover, some food-matrices have acidic or alkaline pH in aqueous solutions, which critically affect the dissolution property and fates of the ZnO NPs [36,37]. Hence, the solubility of food additive ZnO NPs can be influenced by the interaction between ZnO NPs and many food components present in foods or diverse bio-matrices in the body. Our previous study demonstrated that the solubility of ZnO NPs (61 nm of constituent particle size, 261 nm of hydrodynamic diameter, 50 μg/mL) in DW was 1.2%, but increased up to 39.4%, 30.1%, 49.2%, and 90.9% in a coffee mix solution, skim milk solution, milk, and sports drink after incubation for 24 h, indicating the effect of the interactions between the ZnO NPs and food-matrices on the solubility [36]. It is worth noting that the food-matrices were used without dilution for the solubility experiments and the pH of the coffee mix solution, skim milk solution, milk, and sports drink were 6.2, 6.9, 6,9, and 3.3, respectively, suggesting that the pH is not the only factor affecting the dissolution properties of the ZnO NPs. Increased solubility of the ZnO NPs dispersed in milk compared to that in DW was also reported using in vitro simulated digestion systems after 12–24 h, emphasizing the effect of food media on the solubility [30]. However, they only presented the solubility as ppm levels, and not as % levels, thereby limiting direct comparison with other results. Meanwhile, the addition of food stimulants such as starch, milk, oil, and a mixture of the three did not change the solubility of the ZnO NPs in the simulated digestion fluids [31]. The solubility of the ZnO NPs was not affected by the saccharide-based food-matrices, showing 0.7% and 0.2% of solubilities in a 10% honey and 5% sugar mixture, respectively, versus 0.2% in DW [38]. No remarkable effect of the ZnO NPs dispersed in food-matrices (1 mg/mL) including albumin, casein, zein, and glucose versus the ZnO NPs suspended in DW, on the solubility of ZnO NPs was demonstrated in the simulated digestion systems, although the solubility slightly increased in the DW and intestinal fluid in the presence of matrices [29]. The discrepancy of the results seems to be related to the amounts and types of food-matrices used. Moreover, the matrix effect on the solubility seems to be low under extreme conditions where the solubility of ZnO is minimal or almost 100% such as saliva or gastric juice. On the other hand, the solubilities of ZnO NPs in the cell culture minimum essential medium (MEM) were 18.0% and 24.8% after 0.5 h and 24 h, respectively, indicating a role of the interactions between the ZnO NPs and bio-matrices in the solubility because the pH of the cell culture MEM was neutral (7.4) [36].

The solubility results under different conditions are summarized in Table 1. The dissolution properties of the ZnO NPs in processed foods and biological systems must therefore be evaluated and can be important factors in understanding their fates and potential oral toxicity. 

## 3. Interactions between ZnO NPs and Bio- or Food-Matrices

### 3.1. NP Interactions with Bio-Matrices

NPs after oral intake encounter harsh biological environments such as the GI tract where low pH, many enzymes, diverse bio-matrices, and immune systems are present. Interactions between the ZnO NPs and the matrices occur to some extent, which may cause changes in the physicochemical properties, fates, biological responses, and oral toxicity of the ZnO NPs. The interactions between the ZnO NPs and bio-matrices and their impact on biological responses have recently been explored [18,40]. Indeed, biological fluids such as gastric, intestinal, plasma, and body fluids contain many proteins, enzymes, lipids, saccharides, minerals, and other minor components. The ZnO interactions with the simulated gastric, intestinal, and plasma fluids were demonstrated by changes in the hydrodynamic diameters and zeta potentials of ZnO as well as by the increase or decrease in the fluorescence quenching ratios of proteins upon incubation time [26]. The fluorescence quenching ratios of proteins in the presence of NPs can be used as an indication of thee structural conformational changes of proteins [26,41,42]. Further extended ex vivo study showed that a larger amount and number of rat plasma proteins were adsorbed on the surface of the ZnO NPs (28 nm) than the bulk ZnO (290 nm), as analyzed by one- and two-dimensional gel electrophoresis [26]. Proteomic analysis revealed that serum albumin and fibrinogen were the most strongly interacted plasma proteins with ZnO regardless of the particle size, whereas complement C, a part of the complementary system, was only adsorbed on the ZnO NPs, suggesting different biological interactions depending on the particle size [27]. Meanwhile, Shim et al. showed no significant effect of the size (20 nm, 100 nm) or surface charge (+,−) of the ZnO NPs on the numbers of rat blood and brain proteins adsorbed on the ZnO NPs [43], which seems to be related to the similar characteristics of NPs in the size below 100 nm.

Kathiravan et al. and Bhunia et al. reported the formation of a stable BSA-ZnO NP corona associated with the conformational change or unfolding of BSA interacting with ZnO NPs [44,45]. Bukackova and Marsalek also explored the structural change of BSA adsorbed on the ZnO NPs, especially in terms of α-helix structure by circular dichroism (CD), a spectroscopy method measuring the secondary structure of proteins [46]. Sasidharan et al. demonstrated that the electrostatic force of attraction was determined to be involved in the adsorption of BSA on ZnO NPs [47]. In the same context, Bhogale et al. applied fluorescence quenching, UV–Vis absorption spectroscopy, CD, and resonance light scattering spectroscopy techniques to determine the mechanism involved in the ZnO NP interaction with BSA [41]. They showed that ZnO NPs form a ground state complex with BSA by spontaneous binding processes including hydrogen bonding and van der Waals interactions, which leads to fluorophore quenching and the conformational change in BSA. Hence, the interactions between the ZnO NPs and BSA are likely to be reversible under certain environments. On the other hand, Singh et al. demonstrated that the morphology (tetrapodal or spherical shape) of ZnO can be a critical factor preserving the functionality of proteins in the ZnO–protein corona [48]. Giannousi et al. also showed that ZnO nanoflowers with sharp edges exhibited a higher amyloid degradation rate in the BSA and human insulin model proteins than ZnO NPs [49], which requires further extended investigation on a wide range of bio-matrices and NP types.

### 3.2. NP Interactions with Food-Matrices

The interactions between the ZnO NPs and food-matrices have not been relatively well investigated, although food additive ZnO is directly added to processed foods containing diverse matrices. Saccharide is one of the most abundant components in foods. ZnO interactions with various types of saccharide-based food-matrices were quantitatively analyzed including acacia honey composed of several saccharides (~4.24% fructose, ~2.96% glucose, ~0.01% sucrose, and ~0.01% maltose) and other trace nutrients (amino acids and minerals), sugar mixtures (equivalent amounts of fructose, glucose, sucrose, and maltose), and monosaccharide solutions (fructose and glucose, respectively) by high performance liquid chromatography [38]. The results showed concentration-dependent interactions between the ZnO NPs and saccharides. Among them, the glucose in acacia honey and sugar mixtures was found to interact with the ZnO NPs most actively, although fructose is the most abundant saccharide in the honey. On the other hand, 5% fructose more strongly interacted with the ZnO NPs than 5% glucose in the absence of other components, suggesting that minor nutrients and interactions between saccharides can also interactively affect the interactions. Thus, the interactions between the ZnO NPs and food-matrices in processed foods cannot simply be predicted but rather, multiple and complexed interactions are involved.

Food protein–ZnO NP interactions were also investigated with complex protein food-matrices, skim milk (~35% casein, ~50% lactose, lipids, calcium, and other trace nutrients), and simple a protein matrix, casein, showing significant changes in the hydrodynamic diameters and zeta potentials of ZnO NPs, an increase in the fluorescence quenching ratios of the proteins, and the structural conformational changes of the proteins by the interactions [42]. The conformational changes of the skim milk and casein that interacted with the ZnO NPs were demonstrated by CD; deformation of the α-helical bands slightly occurred after incubation with the ZnO NPs for 1 min, and no evident α-helical bands were observed after NP incubation for 168 h, clearly indicating the deformation or conformational change in the proteins [42]. Hence, ZnO–protein interactions can lead to conformational changes of proteins to some extent, which may affect the biological functions of proteins or NP toxicity. However, the interaction between ZnO NPs and food proteins (skim milk, casein, albumin from chicken egg, and zein from corn) had no effect on the digestion efficacy of the proteins in the simulated gastric fluid containing a digestive enzyme pepsin [29,42]. These results imply that the interactions may induce changes in the two-, three-, or four-dimensional structure of proteins, but not completely deform the protein structures. Further extended study revealed that the interactions between ZnO NPs and food proteins did not influence the in vitro cellular uptake and intestinal transport of ZnO NPs using in vitro intestinal barrier models, except for the ZnO interaction with albumin from chicken egg, which showed an enhanced cellular uptake and intestinal transport amount of ZnO NPs [29,42]. Saptarshi et al. pointed out that the adsorbed proteins on NPs may promote the translocation of NPs across cellular barriers, enhancing their cellular uptake, although most studies have been performed in in vitro cell culture systems up to date [50].

In this context, various dispersing agents for NPs are based on bio- or food-matrices. Indeed, glucose, BSA, fetal bovine serum (FBS), carboxymethyl cellulose, and other bio-or food-matrices have been extensively used to disperse NPs for biological applications. A decrease in the hydrodynamic diameters of ZnO NPs in the presence of such dispersants was generally observed, inhibiting the formation of the agglomeration or aggregation of NPs [51,52]. Meißner et al. demonstrated that ZnO NPs in the presence of lecithin or BSA/serum prevented agglomeration in phosphate buffer saline or cell culture Dulbecco’s modified Eagle’s medium due to the adsorption of lecithin or proteins on the particle surface [53]. This aspect is important to evaluate the biological responses of NPs as they are. On the other hand, the antioxidant activity and toxicity of ZnO NPs in the presence of polyphenols and vitamins were investigated, showing an improved seed germination rate and synergistic toxicity, respectively [54,55]. Biological responses were only focused, and the interactions were not qualitatively and quantitatively determined in these studies. Based on the current available information, the ZnO NPs actively interact with bio- or food-matrices in in vitro systems (Table 2), but the direct extrapolation of the in vitro results for in vivo biological systems remains a challenge, requiring further extensive investigation.

## 4. Fates of ZnO NPs in Biological- and Food-Matrices

The most critical point to be considered for the toxicity of food additive ZnO NPs is that the particles are present in intact nano-sized particles, agglomerates/aggregates, or decomposed/ionic forms when they are applied in foods and orally taken. If the ZnO NPs do not contain a fraction of NPs (e.g., NPs form strong aggregates/fused particles or quickly dissolve/degrade into ions/molecules), extended toxicity evaluation recommended for NPs is not further necessary, rather a guidance for conventional materials is sufficient [56]. In other words, nanospecific considerations for toxicity evaluation are not required for materials that do not further possess the typical physicochemical properties of NPs such as a large specific surface area to volume ratio and high reactivity under physiological GI conditions [56]. As above-mentioned in “Section 2. Dissolution properties of ZnO NPs”, ZnO NPs easily dissolve into Zn ions in acidic and biological environments, although their dissolution levels highly depend on the environmental conditions. In this case, the toxicity and biological responses of the ZnO NPs are not only associated with the ZnO particles, but also with the released Zn ions to some extent. Hence, the fate determination of ZnO NPs is of importance in understanding their potential toxicity and toxicity mechanism.

### 4.1. Methodological Approaches for Fate Determination of ZnO NPs

In the actual state, the determination and detection of ZnO NPs as particles without any deformation in processed foods and biological systems are challenging. Inductively coupled plasma (ICP)-atomic emission spectroscopy (AES), ICP-mass spectrometry (MS), and atomic emission spectroscopy are widely applied to determine metallic NPs. However, these methods require harsh digestion conditions including acid and heat treatments to completely digest the organic matrices, thereby leading to Zn release from the ZnO particles. Moreover, the instruments are designated to qualitatively and quantitatively measure the atomic concentrations, and thus the total ionized Zn levels can be determined after Zn ion release from the ZnO particles [57]. Recently, single particle (SP)-ICP-MS has become a powerful technique to quantitatively determine the particle fates of NPs [58]. The detection and characterization of ZnO NPs in wastewater, during drinking water treatment, and in the simulated gastric fluid were carried out by SP-ICP-MS, showing high portions of dissolved Zn ions compared to the intact ZnO NP forms [59,60,61]. Nevertheless, SP-ICP-MS is not easily accessible and requires a high-cost and optimization process. In addition, most of the research on the detection and characterization of the ZnO NPs has been conducted in simple aqueous water or simulated fluids up until now, but not in complex food and in vivo biological systems.

**Figure 2:**
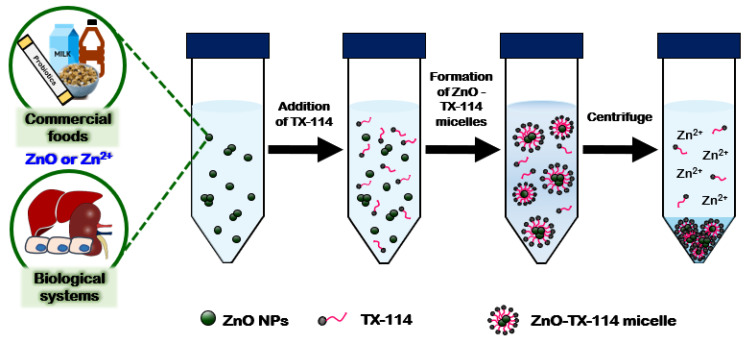
The schematic illustration of the Triton X-114 (TX-114)-based cloud point extraction (CPE) approach for the fate determination of ZnO NPs in commercial foods and biological systems.

A surfactant, Triton X-114 (TX-114)-based cloud point extraction (CPE) approach was recently developed to detect ZnO NPs as intact particle forms in complex systems such as commercial foods and human intestinal cells [37]. The CPE methods have been previously applied for ZnO separation in environmental water where the minimum organic matrices are present [62]. This approach was also reported for the detection of Al or Zn ions in foodstuffs after filtering, acid digestion, or dry-ashing treatment to remove the organic matrices, aiming at determining the total Zn levels [36,63,64]. As above-mentioned, these pre-treatments are not suitable for ZnO detection as intact particle forms. The principle of the CPE is that NPs with zeta potentials close to zero are captured in TX-114-based micelles, and then, the captured NPs are separated into the precipitates after centrifugation. Meanwhile, ionized Zn ions are present in the supernatants after CPE treatment, followed by centrifugation, thereby separating the ZnO particles from the released Zn ions (Figure 2). Both separated particles in the precipitates and ions in the supernatants by CPE can then be analyzed by ICP-AES after the acid and heat treatments. Therefore, intact particle forms can be captured and detected by the CPE approach.

### 4.2. Fates of ZnO NPs in Biological Systems and Processed Foods

**Figure 3:**
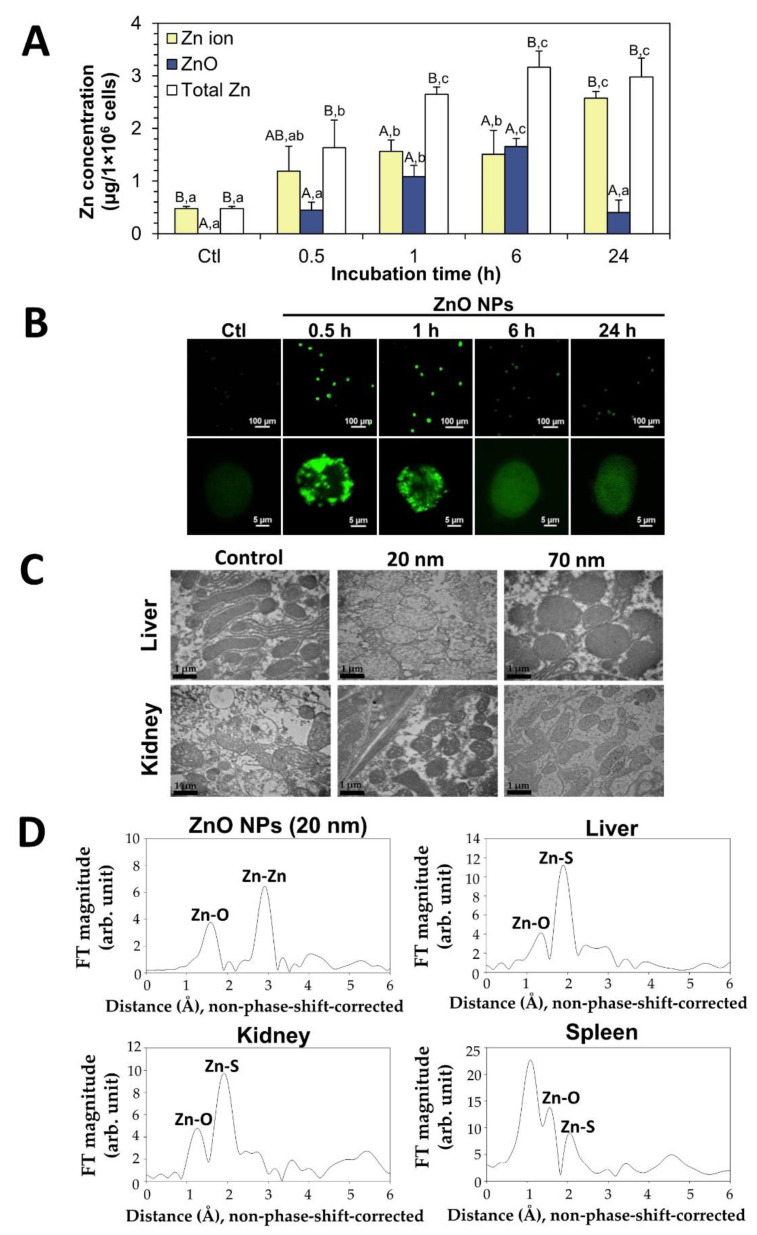
The fates of the ZnO NPs in the human intestinal Caco-2 cells, as determined by (**A**) cloud point extraction (CPE) and inductively coupled plasma-atomic emission spectroscopy (ICP-AES) and by (**B**) a Zn-sensitive fluorescence probe [36]. Different upper case letters (A, B) indicate significant differences between the Zn ions, ZnO, and total Zn in the same incubation time, performed by one-way analysis of variance with Tukey’s test in Statistical Analysis Software (version 9.4) (*p* < 0.05). Different lower case letters (a, b, c) indicate significant differences between the different incubation times in the same sample (*p* < 0.05). (**C**) Transmission electron microscopy (TEM) images and (**D**) X-ray absorption spectroscopic (XAS) spectra of the tissues after a single dose oral administration of ZnO NPs (20 nm) in rats at 24 h post-administration [68].

The toxicity mechanism of ZnO NPs in biological systems cannot be simply understood because they dissolve in aqueous biological fluids to a certain degree. ZnO NPs have been well reported to be internalized into cells by the endocytosis mechanism, an energy-dependent particle uptake, localized in organelles such as lysosomes, and finally decomposed into Zn ions [65,66,67]. The intracellular fate of the ZnO NPs was determined in human intestinal Caco-2 cells by a TX-114-based CPE, showing that both ZnO NPs and Zn ions were present after incubation for 0.5–24 h [36]; Zn ions were primarily present at 0.5 h, both ZnO NPs and Zn ions were present at similar levels at 1–6 h, and most forms detected were Zn ions at 24 h (Figure 3A). This result implies that ZnO NPs can be internalized into cells in both their particle and ionized forms, and then slowly and completely decompose into Zn ions upon the incubation time inside the cells. The intestinal transport fate of the ZnO NPs was also investigated by CPE using an in vitro Caco-2 monolayer and follicle-associated epithelial models and ex vivo everted small intestinal sacs [37]; the major transport fate of the ZnO NPs was as Zn ions, but a small portion of the particle forms was also detected in both the in vitro and ex vivo models. The in vivo fate of ZnO NPs can be predicted in accordance with their dissolution properties in the GI tract, as mentioned in Section 2. Therefore, the toxicity of ZnO NPs is surely related to the Zn ion release from the particles to some extent, but the potential toxicity that resulted from the ZnO NPs must also be considered after long-term exposure.

Another important point to be addressed is whether the released Zn ions from the ZnO NPs remained in their ionized forms or not, without re-forming the crystalline phase with the other ligand molecules massively present in the body. Gilbert et al. demonstrated that ZnO NPs can be taken up by cells in the particle form and completely dissolve inside the cells, generating intracellular Zn ions complexed with molecular ligands by high resolution X-ray spectromicroscopy and high elemental sensitive X-ray microprobe analysis [68]. A time-dependent increase in the cellular uptake of ZnO NPs after 0.5–6 h in human intestinal Caco-2 cells was reported by CPE treatment followed by ICP-AES analysis (Figure 3A), whereas their maximum uptake was at 0.5–2 h, as determined by a Zn-sensitive fluorescence probe (Figure 3B) [37]. Hence, the released Zn ions from the particles in the cell culture medium seem to be easily taken up by cells at the initial times of incubation, and the internalized ZnO NPs could decompose into Zn ions inside the cells. The discrepancy between Figure 3A,B can be explained by the complex formation between the ionized Zn and the cellular molecular ligands upon incubation time when the cellular uptake was measured by the Zn-sensitive probe at 6–24 h, thereby decreasing the Zn-sensitive fluorescence intensity. Baek et al. reported that no particle form was detected in the liver and kidney by transmission electron microscopy (TEM) (Figure 3C), and new Zn–S bonds were found in the tissues by X-ray absorption spectroscopy (XAS) (Figure 3D) after a single-dose oral administration of ZnO NPs in Sprague-Dawley rats, suggesting the role of the interaction between the released Zn ions and the S-ligand-containing proteins in their uptake and distribution [69]. Indeed, cysteine-containing metallothionein is known to be involved in the Zn uptake into the cells and tissues [70,71]. The formation of the re-crystalline phase of the Zn–carbonate or Zn–phosphate in the intestine after complete ionization of the ZnO in the gastric juice must also be considered. Comprehensive consideration including the degree of ionization, interactions, and re-crystallization is necessary for understanding the in vivo fate of ZnO NPs.

On the other hand, manufacturing processes such as heating, cooling, mixing, and coating can also influence the particle or decomposed fates of NPs. Until now, little information is available on the presence and fate of ZnO particles in foods. The ionic or particle fates of ZnO particles in Zn-fortified commercial foods where ZnO additions are indicated as labeled were investigated using the TX-114-based CPE approach [37]. The results demonstrated that most of the ZnO was present as particles in the powdered processed foods such as chocolate powder, powdered probiotics, and cereals, whereas the major fate of ZnO was as Zn ions in liquid beverages such as functional peptide beverages and fruit juice, where the pH ranged from 3 to 5. These findings suggest that the fate of ZnO is highly dependent on the manufacturing process and matrix types. The dissolution property, pH, and ZnO–matrix interactions in complex food-matrices should also be considered to determine their fate in processed foods. Grasso et al. recently determined the ZnO NPs in canned seafood by SP-ICP MS, demonstrating that ZnO NPs in the size range of 63.1 to 78.6 nm were detected at 0.003–0.010 mg/kg [17]. This result suggests a potential bioaccumulation of ZnO NPs in marine organisms, although the estimated daily intake of ZnO NPs by seafood for adults is low (0.010–0.031 μg/kg body weight). The possibility that the crystal formation of ZnO particles from Zn ions, not attributed to ZnO uptake in marine organisms, cannot be completely excluded. The mechanism for the formation of ZnO NPs in living organisms is required to be elucidated and an investigation across a wide range of seafood is necessary.

## 5. Cytotoxicity and Oral Toxicity of ZnO NPs Interacted with Bio- or Food-Matrices

**Figure 4:**
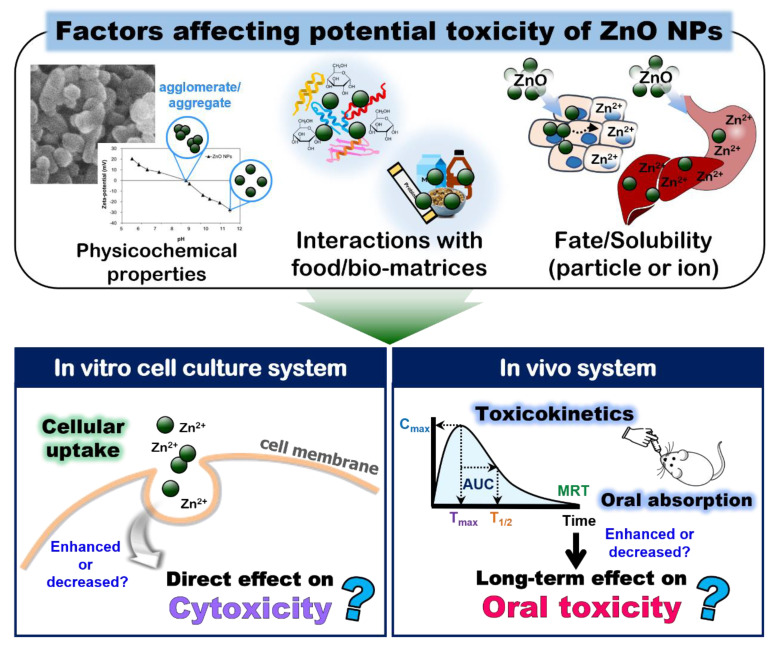
The schematic illustration of the cytotoxicity and oral toxicity affected by the physicochemical properties, interactions with bio- or food-matrices, fate, and the solubility of ZnO NPs.

The toxicity of ZnO NPs is generally accepted to be associated with the released Zn ions from the particles and the generation of reactive oxygen species (ROS), suggesting an important role of the dissolution levels in the toxicity under physiological conditions [72,73,74]. The cytotoxicity of ZnO with respect to particle size has been intensively explored [75,76]. Many reports have demonstrated its size-dependent cytotoxicity in terms of cell growth inhibition, cell death, and ROS generation, showing a higher toxicity of small-sized ZnO NPs than larger-sized particles [77,78,79]. A relatively high toxicity of ZnO compared with the most widely applied food additive NPs such as silicon dioxide (SiO_2_) and titanium dioxide (TiO_2_) has been reported, probably related to the high toxicity of Zn [80]. On the other hand, the cytotoxicity of ZnO NPs can also be affected by interactions with the bio- or food-matrices. ZnO NPs (~70 nm of constituent particle size, highly aggregated) dispersed in BSA or FBS significantly inhibited the cell proliferation of lung epithelial A549 cells compared with the ZnO NPs in the cell culture medium, which was attributed to enhanced cellular uptake [51]. Anders et al. reported that FBS enhances the dispersion stability of ZnO NPs (~10 nm, ~300 nm of hydrodynamic diameter) by increasing or reducing their cytotoxicity, depending on the cell line types (suspension or adherent cells) [81]. Janani et al. recently showed that ZnO NPs (65 nm of hydrodynamic diameter) that interacted with BSA exhibited a lower toxicity than that of pristine ZnO NPs in environmental model systems of plant, bacteria, algae, and crustaceans in terms of ROS generation, lipid peroxidation, and chromosomal aberrations [82]. Precupas et al. showed that BSA adsorption onto the NPs was spontaneous and enthalpy-controlled, which decreased in the order of ZnO > SiO_2_ > TiO_2_, whereas the structural stability of BSA decreased in the order of the presence of TiO_2_ > SiO_2_ > ZnO [83]. In other words, they demonstrated the correlation between the increased enthalpic character of the interaction and decreased structural stability of BSA, which also causes biological alterations such as DNA strand breaks and cell death. Da Silva et al. reported that ZnO NPs decreased the lactate dehydrogenase (LDH) activity due to the concentration-dependent LDH adsorption onto the particles and the LDH inhibition by the interaction with dissolved Zn from ZnO [84]. It is strongly likely that the interactions between the ZnO NPs and biomatrices can modulate in vitro biological responses and the cytotoxicity of ZnO NPs (Figure 4), although the current information is not sufficient to conclude on the interaction effect.

Food-grade albumin from chicken egg reduced the hydrodynamic diameter of ZnO NPs (~78 nm of constituent particle size, ~375 nm of hydrodynamic diameter), causing increased cell growth inhibition, membrane damage, and ROS generation at high concentrations of ZnO NPs (125–500 μg/mL) compared with the ZnO NPs in the DW or cell culture medium in the human intestinal Caco-2 cells [29]. The study also revealed that the increased cytotoxicity was attributed to the enhanced cellular uptake of ZnO NPs in the presence of food albumin [29]. Meanwhile, it is interesting to note that the ZnO NP interaction with casein or zein significantly reduced the ZnO toxicity compared with the ZnO in DW, implying an important role of the interaction between ZnO and the food components in the toxicity [29]. Contradictory results were obtained with highly aggregated ZnO NPs (~25 nm of constituent particle size and ~1957 nm of hydrodynamic diameter) that interacted with casein, showing no significant effect on the cytotoxicity in the same Caco-2 cell line [42]. The discrepancy can be explained by different agglomerate/aggregate states between the ZnO NPs used in these two studies. The interaction effect on the cytotoxicity of the highly aggregated ZnO NPs seems to be minor. Indeed, the cytotoxicity of the ZnO NPs with a ~25 nm constituent particle size and ~1999 nm hydrodynamic diameter was not affected by their interaction with saccharides [38]. Hence, the physicochemical properties and fates of the ZnO NPs are also critical factors affecting the cytotoxicity caused by the interactions between the ZnO NPs and matrices. Cao et al. showed the synergistic effects of ZnO NPs (~150 nm of hydrodynamic diameter) and palmitic acid on the cytotoxicity in Caco-2 cells, which were related to increased mitochondrial ROS, whereas the co-exposure of ZnO NPs and free fatty acids did not increase the ROS generation [85]. Wang et al. showed that vitamin C increased the cytotoxicity of ZnO NPs compared with the ZnO NPs only, and the combined synergistic toxicity was also found after repeated oral administration in mice in terms of the injury of the liver and kidney [86]. Therefore, a toxicity evaluation of the NPs used in foods is required to be performed in a complex food system (Figure 4). More extended data including in vivo experiments are needed to correlate the interaction effects on the toxicity of ZnO NPs.

On the other hand, the toxicokinetics and oral absorption of ZnO NPs have been reported to be affected by the interactions. Among the saccharides including honey, a sugar mixture, fructose, and glucose, the ZnO NPs in glucose significantly enhanced the oral absorption (13.4%), followed by the order of ZnO NPs in fructose (9.6%) > sugar mixture (8.2%) > DW and honey (~7.0%) after a single-dose oral administration in rats [38]. ZnO NPs that interacted with glucose or fructose also showed a prolonged half-life and mean residence time in rats compared with the ZnO NPs in DW [38]. When the toxicokinetics of the ZnO NPs in glucose and albumin, the most interacted saccharide and food protein with ZnO NPs, respectively, were compared, the ZnO NPs in glucose had a significantly enhanced (~2.1 fold) oral absorption and increased (~1.8 fold) half-life compared to the ZnO NPs in DW, whereas no statistical differences in the toxicokinetic parameters were found between the ZnO in albumin and ZnO in DW [29]. A repeated oral toxicity study of the ZnO NPs interacting with albumin or glucose in rats for 14 d revealed no effect of the interactions on oral toxicity based on changes in the body weight, food intake, and water consumption, hematological and serum biochemical parameters, and histopathological examination [29]. The results suggest that the interactions between the ZnO NPs and food-matrices could directly affect the in vitro biological responses and cytotoxicity but may not influence the in vivo oral toxicity. It is interesting to note that the enhanced oral absorption of ZnO NPs in glucose did not cause a higher oral toxicity than the ZnO NPs in DW, implying that an increased absorption amount of ZnO NPs might not be enough to increase the oral toxicity. Hence, care and caution are needed to interpret the in vitro data. However, the oral toxicity study by Jung et al. was performed for a relatively short period of 14 d [29], thereby needing more long-term in vivo oral toxicity experiments. On the other hand, it is possible that toxicological effects occur at microscopic levels such as gene expression and transcriptomic responses, and not at macroscopic levels including the blood/serum biochemical parameters and histopathological changes. Indeed, Yu et al. reported that gene expression profiles in the livers, analyzed by next-generation sequencing, were differently affected by the fate (ion or particle) and particle size (nano or bulk) of the ZnO following 14 d repeated oral administration in rats [27]; heme/metal binding and cytochrome P450 were upregulated by ZnO NPs, whereas the metabolic, catabolic, oxidation–reduction process, and heme/cytochrome P450 were affected by the Zn ions. Only the cellular process was slightly influenced by the bulk ZnO. These results imply that the unexpected potential toxicity caused by ZnO NPs must also be also considered after long-term exposure (Figure 4). Table 3 summarizes the cytotoxicity and oral toxicity caused by the interactions between the ZnO NPs and the bio- or food-matrices.

## 6. Conclusions

Food additive ZnO NPs are added in complex food-matrices and orally taken, and thus a comprehensive understanding of their characteristics and fates in processed foods and in the body is required to understand and predict their potential toxicity and toxicity mechanism. ZnO NPs can dissolve into Zn ions in food-matrices and the GI fluids to some extent, related to the low pH and NP interactions with the matrices. However, the question as to whether ZnO NPs completely decompose into Zn ions or re-form a crystalline phase with other molecular ligands present in the intestine and tissues remains to be answered. The formation of ZnO NPs in living organisms in nature also needs to be elucidated. The detection of ZnO NPs as intact particle forms in complex bio- or food-matrices is also challenging. The toxicity and biological responses of ZnO NPs seem to be associated with both the ZnO particles and released Zn ions. The current available information suggests that the interactions between the ZnO NPs and bio- or food-matrices can modulate the cytotoxicity and cellular uptake of ZnO NPs in cell lines. The interactions can enhance the oral absorption of ZnO NPs after a single-dose administration, but may not affect acute oral toxicity. However, the increased absorption effect on the oral toxicity of ZnO NPs after long-term use must be confirmed. Future perspectives that remain to be answered to ascertain the safety aspects of food additive ZnO NPs are listed in Table 4.

## Figures and Tables

**Table 1:** The dissolution properties of the ZnO NPs under different conditions.

Physicochemical Properties	Conditions	Concentrations	Solubilities	Reference
28 nm ^1^, 1976 nm ^2^ 290 nm ^1^, 3453 nm ^2^	Simulated gastric (pH 1.5) fluid	5 mg/mL	24.5%	[26]
	Simulated intestinal (pH 6.8) fluid		0.2%	
	Simulated plasma fluid		2.8%	
	Rat-extracted gastric fluid	5 mg/mL	~12%	
	Rat-extracted intestinal fluid		~9%	
	Rat-extracted plasma fluid		~2%	
86 nm ^1^, 401 nm ^2^ 268 nm ^1^, 604 nm ^2^	In vivo rat gastric fluid (oral administration)	100 mg/kg	~12%	[27]
<50 nm ^1^ <100 nm ^1^	Neutral pH (7.0)	0.5 mg/mL	1.87–2.13%	[25]
	Low pH (1.5)		93.6–97.0%	
78 nm ^1^, 375 nm ^2^	DW	5 mg/mL	0.1%	[29]
	Cell culture MEM		0.5–0.7%	
	Simulated saliva	5 mg/mL	~0.1%	
	Simulated gastric fluid		~96%	
	Simulated intestinal fluid		~4%	
	Simulated saliva + gastric fluid	5 mg/mL	95%	
	Simulated saliva + gastric + intestinal fluids		25%	
15–70 nm ^1^, 180 nm ^2^ 20–350 nm ^1^, 245 nm ^2^	Simulated saliva	209–8338 μg/mL ~93–3706 μg/mL 31–1250 μg/mL	<5%	[31]
	Simulated saliva + gastric fluid		~100%	
	Simulated saliva + gastric + intestinal fluid		13–34%	
20 nm × 100 nm ^1^ (rod type), 1636 nm ^2^ ~200 nm ^1^, 1107 nm ^2^	Simulated gastric fluid	30 μg/mL ~11 μg/mL	10.6–14.2%	[32]
	Simulated gastric + intestinal fluid		1.72–1.89%	
40 nm ^1^	Water	100 μg/mL	2.2%	[39]
	Cell culture RPMI 1640		2%	
	Cell culture RPMI 1640 + FBS		1%	
	Artificial lysosomal fluid		98.1%	
61 nm ^1^, 261 nm ^2^	DW	50 μg/mL	1.2%	[37]
	Coffee mix solution		39.4%	
	Skim milk solution		30.1%	
	Milk		49.2%	
	Sports drink		90.9%	
	Cell culture MEM		18.0–24.8%	
25 nm ^1^, 1999 nm ^2^	DW	5 mg/mL	0.2%	[38]
	10% honey		0.7%	
	5% sugar mixture		0.2%	

^1^ Constituent particle size measured by scanning or transmission electron microscopy. ^2^ Hydrodynamic diameters measured by dynamic light scattering. Abbreviations: DW, distilled water; MEM, minimum essential medium; RPMI, Roswell Park Memorial Institute; FBS. fetal bovine serum.

**Table 2:** A summary of the interactions between the ZnO NPs and bio- or food-matrices.

Interaction Matrices		Results	Reference
ZnO	Matrix Types		
Bulk ZnO (290 nm ^1^) ZnO NPs (28 nm ^1^)	Simulated gastric fluid Simulated intestinal fluid Simulated plasma fluid	Hydrodynamic diameters, zeta potentials, and fluorescence quenching ratios of proteins changed.	[26]
	Rat plasma proteins	Serum albumin and fibrinogen strongly interacted with both the bulk ZnO and ZnO NPs, but complement C was only adsorbed onto the ZnO NPs.	
ZnO NPs (7.5 nm ^1^)	BSA	ZnO NPs formed ground state complex with BSA.	[41]
+, −-charged ZnO NPs (20 nm ^1^, 100 nm ^1^)	Rat brain proteins Rat plasma proteins	Size or surface change of ZnO NPs did not affect the number of proteins adsorbed.	[43]
Colloidal ZnO NPs (2.5 nm ^1^)	BSA	Interaction between ZnO NPs and BSA led to conformational change of BSA.	[44]
ZnO NPs (15–20 nm ^1^)	BSA	Formation of a stable BSA–ZnO NP corona was associated with conformational change/unfolding of BSA.	[45]
ZnO NPs (68.1 nm ^1^, 78.8 nm ^1^)	BSA	BSA adsorbed onto ZnO NPs showed α-helical structural change.	[46]
Colloidal ZnO NPs (65 nm ^2^)	BSA	Electrostatic force of attraction was involved in BSA adsorption onto ZnO NPs.	[47]
Tetrapodal ZnO (15 μm ^1^)Spherical ZnO NPs (100 nm ^1^)	Insulin	Tetrapodal ZnO preserved the polarity and surface charge distribution of insulin.	[48]
ZnO nanoflower (168 nm ^1^) ZnO@PEG NPs (40 nm ^1^)	BSA Human insulin	ZnO nanoflower showed higher amyloid degradation rate in both proteins.	[49]
ZnO NPs (25 nm ^1^)	10% honey 5% sugar mixtures 5% monosaccharide solutions	ZnO NPs actively interacted with glucose in the honey and sugar mixtures, but they most strongly interacted with fructose among the monosaccharide solutions.	[38]
ZnO NPs (25 nm ^1^, 1957 nm ^2^)	Skim milk Casein	The hydrodynamic diameters, zeta potentials, fluorescence quenching ratios of protein, and α-helical protein structure changed, but not the digestion efficacy.	[42]
ZnO NPs (78 nm ^1^, 375 nm ^2^)	Albumin Casein Zein	Primary structural stability or digestion efficacy of proteins were not affected by the interactions.	[29]
ZnO NPs (234 nm ^2^)	Lecithin BSA/serum	Adsorption of proteins on the surface of ZnO NPs prevented agglomeration.	[53]

^1^ The constituent particle size measured by the scanning or transmission electron microscopy. ^2^ Hydrodynamic diameters measured by dynamic light scattering. Abbreviations: BSA, bovine serum albumin.

**Table 3:** A summary of the cytotoxicity and oral toxicity of the ZnO NPs that interact with the bio- or food-matrices.

Interaction Matrices		Models	Results	Reference
ZnO	Matrix Types			
ZnO NPs (70 nm ^1^)	BSA FBS	A549 cell	ZnO NPs dispersed in BSA and FBS showed a high cell proliferation inhibition associated with enhanced cellular uptake.	[51]
ZnO NPs (10 nm ^1^)	FBS	Jurkat T cell Hut-78 T cell T-47D cell LNCaP cell	Interactions between ZnO NPs and FBS increased or decreased the cytotoxicity depending on cell lines.	[81]
ZnO NPs (65 nm ^2^)	BSA	*p. aeruginosa/S. aureus* (bacteria) *C. pyrenoidsa* (algae) *Daphnia sp.* (crustacean) *A. Cepa* root cells (plant)	ZnO that interacted with BSA reduced the ROS generation, lipid peroxidation, and chromosomal aberrations.	[82]
ZnO NPs (158 nm ^1^)	BSA	A549 cell	BSA adsorption onto the ZnO NPs was spontaneous and enthalpy-controlled, decreasing the structural stability of BSA and causing biological alterations.	[83]
ZnO NPs (106 nm ^1^, 101 nm ^1^)	LDH	Complete Ham’s F12 medium	ZnO NPs decreased the LDH activity due to LDH adsorption onto the ZnO and interaction with dissolved Zn ions	[84]
ZnO NPs (78 nm ^1^, 375 nm ^2^)	Albumin Casein Zein Glucose	Caco-2 cell SD rat	- ZnO NPs that interacted with albumin caused high cytotoxicity attributed to the high cellular uptake, but oral absorption did not increase - ZnO NPs in casein reduced the cytotoxicity - ZnO NPs in glucose enhanced the oral absorption, but did not affect the oral toxicity	[29]
ZnO NPs (25 nm ^1^, 1957 nm ^2^)	Skim milk Casein	Caco-2 cell	ZnO NPs that interacted with casein did not increase the cytotoxicity.	[42]
ZnO NPs (25 nm ^1^, 1999 nm ^2^)	10% honey 5% sugar mixtures Monosaccharide solutions	Caco-2 cell SD rat	Cytotoxicity of the ZnO NPs was not affected by the interactions with saccharides, but their toxicokinetics and oral absorption increased.	[38]
ZnO NPs (150 nm ^2^)	Palmitic acid Free fatty acids mixture	Caco-2 cell	ZnO NP interaction with palmitic acid, but not with free fatty acid, increased the cytotoxicity related to ROS generation.	[85]
ZnO NPs (25 nm ^1^)	Vitamin C	GES-1 cell Kunming mice	- Vitamin C increased the cytotoxicity of ZnO NPs - Synergistic toxicity was found after repeated oral administration of ZnO NPs plus vitamin C	[86]
Bulk ZnO (268 nm ^1^) ZnO NPs (86 nm ^1^)	-	SD rat	Gene expression profiles in the livers were influenced by the fate and particle size of the ZnO.	[27]

^1^ The constituent particle size measured by scanning or transmission electron microscopy. ^2^ Hydrodynamic diameters measured by dynamic light scattering. Abbreviations: BSA, bovine serum albumin; FBS, fetal bovine serum; LDH, lactate dehydrogenase; SD, Sprague-Dawley.

**Table 4:** The future perspectives to understand and predict the potential toxicity of food additive ZnO NPs.

- Dissolution properties of ZnO NPs in various processed foods and biological systems - Interactions between diverse types of ZnO NPs and a wide range of bio- or food-matrices - Extensive experimental data on the interaction effects on in vitro and in vivo biological systems - Development of reliable methodological approaches for the fate determination of ZnO NPs in matrices - Relationship between fate and the long-term toxicity of ZnO NPs - Comprehensive consideration of the solubility, interactions, and re-crystallization for an understanding of the in vivo fate and toxicity of ZnO NPs - Elucidation on the formation of ZnO NPs in living organisms in nature - Potential toxicity caused by ZnO NPs after long-term exposure

## Data Availability

The data presented in this study are available in the article.
